# The continuum of critical care

**DOI:** 10.1186/s13054-019-2393-x

**Published:** 2019-06-14

**Authors:** Jean-Louis Vincent

**Affiliations:** Department of Intensive Care, Erasme University Hospital, Université Libre de Bruxelles, 808 Route de Lennik, 1070 Brussels, Belgium

**Keywords:** Disease trajectory, Communication, Outcomes

## Abstract

Until relatively recently, critical illness was considered as a separate entity and the intensive care unit (ICU), often a little cut-off from other areas of the hospital, was in many cases used as a last resort for patients so severely ill that it was no longer possible to care for them on the general ward. However, we are increasingly realizing that critical illness should be seen as just one part of the patient’s disease trajectory and how the patient is managed before and after ICU admission has an important role to play in optimizing outcomes. Identifying critical illness early, before it reaches a stage where it is life-threatening, is a challenge and requires a combination of improved and more frequent or continuous monitoring of at-risk patients, staff training to recognize when a patient is deteriorating, a system to “call for help,” and an effective response to that call. Critical care doctors are now widely available 24 h a day for consultation, and many hospitals have rapid response or medical emergency teams composed of staff trained in intensive care and with resuscitation skills who can attend patients on the ward who have been identified to be deteriorating, assess them to determine the need for ICU admission, and initiate further tests and/or initial therapy. Early intensivist input may also be important for patients undergoing interventions that are likely to result in ICU admission, e.g., transplantation or cardiac surgery. The patient’s continuum after ICU discharge must also be taken into account during their ICU stay, with attempts made to limit the longer-term physical and psychological consequences of critical illness as much as possible. Minimal sedation, good communication, and early mobilization are three factors that can help patients survive their ICU stay with minimal sequelae.

## Introduction

The development of critical illness rarely occurs without warning, but is preceded by a series of, often undetected, changes in vital clinical signs over a period of hours [1]. Once admitted to the intensive care unit (ICU), a critically ill patient will receive multiple interventions to help prevent any further deterioration and, hopefully, lead to recovery. Yet in many patients, early deterioration is missed and they are admitted late to the ICU making salvage more difficult and reducing the chances of survival. Even when signs of deterioration are noticed, there is often no system in place to direct the next step and patients may be left waiting while the message moves up the hierarchical ladder to someone more qualified or authorized to act upon it. Once on the ICU, the management received can impact considerably on post-ICU long-term outcomes.

**Fig. 1:**
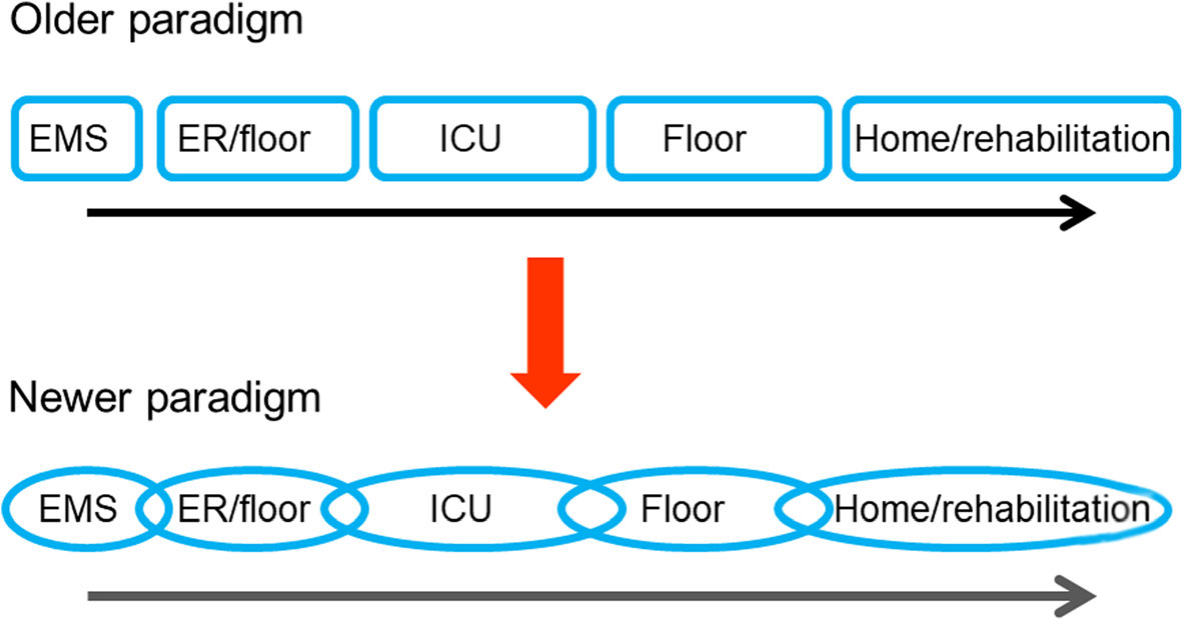
Schematic depicting older and newer paradigms of critical illness, with borders between units and phases becoming less clear cut as collaboration and communication improves during the patient’s trajectory. EMS: emergency medical services; ER: emergency room; ICU: intensive care unit

Critical illness therefore needs to be seen as a continuum, a continuous sequence of interlinked events from the very early moments of illness, through the ICU stay, and into recovery and rehabilitation (Fig. 1). For the purposes of this text, and as is generally still the case in clinical practice, we will cut the continuum into pre-ICU, ICU, and post-ICU situations and consider the events within each phase that may influence the next part of the continuum.

### Pre–ICU

#### Pre-hospital

In terms of pre-hospital management, precise strategies to improve continuity of care and reduce fragmentation will depend on the underlying condition and available emergency medical services. In all cases, rapid appropriate patient management and effective collaboration among the emergency services and receiving hospitals is essential to optimize outcomes. This process should ideally start with the public who are often the first on the scene and can already provide essential elements of first aid and even resuscitation. For example, the importance of bystander cardiopulmonary resuscitation (CPR) in patients with out-of-hospital cardiac arrest has been appreciated for years with multiple studies demonstrating the positive effects of this immediate intervention on short- and long-term outcomes [2, 3]. The so-called chain-of-survival must then continue through more advanced resuscitation by trained emergency services personnel supported by telephone-guidance and telemedicine input if necessary, through patient transfer to the nearest center able to provide the necessary (surgical) interventions and finally to the multidisciplinary hospital team ready to continue management. Each step is facilitated by accurate, repeated exchange of information among all those involved.

Similarly, for patients with stroke, public education is needed to increase awareness of the signs and symptoms of stroke and encourage early calls to the emergency medical services. Rapid patient management in an emergency stroke mobile unit has been associated with more rapid thrombolysis and better short-term outcomes [4]. Indeed, computed tomography (CT) scans can now be obtained in ambulances so that the decision for thrombolysis (in the absence of intracerebral bleeding) can already be made prior to hospital admission—these mobile CT scanners will be increasingly available in the future. Again, a streamlined approach is necessary with the receiving hospital notified in advance of the incoming patient, continuous communication of patient status, and readiness of a multidisciplinary team on arrival to avoid delays waiting in the emergency department.

Pre-hospital trauma management, including major disasters, is perhaps the area where most improvement has been seen in recent years, often as a result of lessons learned from the military arena being transferred to the civilian setting [5]. Rapidly dispatched trauma teams, often including medical personnel, provide early on-site resuscitation and stabilization. Patients are then triaged to the most appropriate center where their arrival is anticipated due to continued communication with the emergency medical services such that appropriate management can be continued and more definitive treatment organized within the hospital.

#### In-hospital, pre-ICU

Traditionally, within the hospital, patient care has, in many ways, been rather fragmented with management passed from one set of carers to another. This is partly a legacy of hospital growth and expansion as new disciplines and departments have developed over the years, often simply being added on to older general ward buildings [6], resulting in a structure of multiple separate parts rather than a single coordinated system. The interfaces between these separate units are where the continuum of care often breaks down with poor communication and collaboration between medical disciplines and staff members resulting in a lack of continuity and poor transfer of information. This in turn facilitates errors in patient management.

Perhaps the most important aspect of in-hospital pre-ICU patient management in terms of the continuum of critical illness is the detection of possible deterioration in patient condition. Much of the “chain of survival” concept has been applied to the pre-hospital management of cardiac arrest, so the “chain of prevention” concept has been suggested to decrease the likelihood and consequences of patient deterioration [7]. Although originally proposed for in-patient cardiac arrest management, the steps—staff education, monitoring, recognition of deterioration, how to “call for help”, and an effective response—can equally apply to all other conditions that may lead to the need for intensive care.

Patient monitoring on the general hospital ward is largely based on intermittent (often infrequent) observations and measurements of simple variables, e.g., blood pressure and temperature, by the most junior nursing staff or assistants. As a result, patient deterioration if it occurs between scheduled measurements may go unnoticed. Repeated staff training at all levels is necessary to ensure that all staff are familiar with possible signs of deterioration. Monitoring of vital signs needs to be performed more regularly, and with the development of smaller, more mobile, connected monitors, this is becoming more feasible without increasing nurse workload [8]. Early identification of deterioration on general hospital wards can help reduce the need for transfer to higher acuity units, reduce hospital lengths of stay and costs, and improve survival rates [9]. In one study of 401 general ward patients requiring ICU admission, each hour of delay in admission was associated with a 1.5% increase in the risk of ICU death and a 1% increase in hospital mortality [10].

Various methods have been developed to alert staff to risk of patient deterioration on the general ward, for example, the Modified Early Warning Score (MEWS) [11] and the National Early Warning Score (NEWS) [12], both of which allocate points when physiological variables deviate from “normal” when measured manually and then use the sum to trigger a specific, often graded response defined according to local protocol. Recently, the Quick Sequential Organ Failure Assessment (qSOFA) score has been proposed to assess the risk of sepsis and potential need for intensive care in patients with suspected infection on the general floor [13, 14]. The presence of at least two of the three score variables (respiratory rate ≥ 22 breaths/min, altered mental status [decrease in Glasgow Coma Scale score of ≥ 1 point from the patient’s normal baseline], or systolic blood pressure ≤ 100 mmHg) should trigger an alert encouraging further evaluation for sepsis, assessment by a sepsis team, and consideration of transfer to an ICU. As monitoring on the general ward increases and becomes more automated, such warning systems will also become automated, triggering an audible or electronic alarm when specific parameters are met. Importantly, all these warning systems are just one means of providing an alert of possible deterioration and will not detect all cases. Clinical judgment and nurse concern must also be acknowledged and all hospital staff should be trained to recognize the signs of deterioration early, and not just to rely on a specific score [8].

Importantly, identification of deterioration alone is of course not sufficient to improve outcomes. A system must be in place to determine how to manage the patient in question. Generally, escalating protocols are used, determined by the degree of deterioration detected and whether the patient’s condition is worsening or getting better. Such systems have to be adapted to the local hospital organization and available facilities. Often an increase in frequency of monitoring and notification of a more senior member of staff are the first steps, followed eventually by a call to some form of rapid response system (RRS) that can attend, assess, and if necessary start treatment of patients on the general hospital ward who have early signs of deterioration in order to prevent further worsening. Transfer to the ICU can then be arranged if necessary. This type of call for a deteriorating patient needs to be differentiated from “code blue” or “crash” calls (for example for cardiac arrests) where specific teams will run to assist with resuscitation. Members of the RRS called to a deteriorating patient outside the ICU will arrive rapidly but without the emergency associated with a code blue call. In some hospitals, the two teams will include the same personnel, but the speed and type of response will be different. Most RRS include a doctor and a physiotherapist (for respiratory distress) or an experienced nurse, or some combination of these, which is why the term RRS is better than medical emergency team, which implies only doctors are included. In our institution, the “code blue team” includes a doctor and one or two nurses, the RRS includes one doctor, and we have a separate phone number for a physiotherapist (all available 24/7). We also have a shock room to which such patients can be admitted for initial resuscitation before transfer to the ICU [15]. The four-bed room is fully equipped for all emergencies and staffed 24 h. Although difficult to study given the nature of the intervention, several reports have suggested that RRS are associated with reduced incidence of in-hospital cardiac arrest, reduced ICU admissions, and improved patient outcomes [16, 17]. When staffed by members of the ICU team, they also help provide continuity of care from the general ward to the ICU.

### In-ICU

Once a patient is admitted to the ICU, successful management should aim not only at patient survival, but also at ensuring good quality of life post-ICU and maximizing the quality of the dying process in patients who succumb [18]. While dealing with the acute present condition, it is therefore important to think about the future for each patient. Multiple ICU treatments and interventions can impact on short- and long-term physical and psychological patient outcomes. Indeed, over recent years, many standard ICU interventions have been shown to have negative effects when used excessively: tidal volumes that are too large [19], too many blood transfusions [20], too much oxygen [21], and too much sedation [22, 23] can all have negative effects on outcomes. Vasoactive agents have been associated with the development of ICU-acquired weakness [24], which may impact on outcomes, although further data are needed to confirm these findings. Other interventions can improve outcomes. For example, early (within 72 h) patient mobilization, facilitated by reduced sedative use, has been associated with better outcomes [25]. Extended (or open) family visiting hours [26, 27] enabling contacts with the patient’s home life and even visits of pets [28] can help limit the psychological impact of an ICU stay. If it is clear that the patient is not going to survive and that further intensive therapy will make no difference to the outcome, then life-sustaining interventions should be stopped and palliative care commenced to ensure the patient dies with dignity [29].

### Post-ICU

The decision to discharge a patient from the ICU should be made carefully as premature discharge is associated with ICU readmission, and possibly with increased mortality [30]. The timing of the discharge as well as patient condition must be taken into account. Out-of-hours discharges are associated with in-hospital death and ICU readmission [31]. Perhaps, the most important aspect of post-ICU care is good communication during patient transfer to the regular ward. Non-intensivist colleagues should be informed of any specific issues that may arise and how to manage them to avoid a further ICU admission. Communication with the patient and their families is also important because the change from the high-intensity monitoring and staffing levels to the general ward can be disconcerting and frightening. A follow-up visit from a familiar member of the ICU staff may help allay some of these concerns. Once discharged from the ICU to the hospital floor, monitoring to detect possible deterioration is again essential, as discussed earlier.

When the patient is discharged home or to a long-term care facility, good communication is again key to prepare the patient, family, and the primary care team for this next phase of the continuum. Post-ICU clinics are becoming more common and may have an important role in improving outcomes as patients and families have many questions, worries, nightmares etc., which can be best answered by someone from the intensive care team. Patients may also have questions related to a clinical trial in which they were enrolled, which again may not be familiar to the primary care physician. Clearly, issues regarding how often and for how long patients should attend these clinics, who they should be staffed by, what tests and assessments should be performed, and what impact they have on long-term outcomes need further study [32]. Increased use of telemedicine may facilitate post-ICU follow-up enabling continued monitoring and virtual follow-up visits in the patient’s home.

## Conclusion

The ICU has developed as a late addition to many hospitals and, in many cases, has failed to become integrated with the rest of the hospital system. Yet the development of critical illness is just one part of a patient’s disease trajectory, and patient management should be streamlined from the very earliest signs of illness through to recovery. Wherever the patient is on their trajectory, they must remain at the center of our preoccupations. Better collaboration, communication, and teamwork between emergency medical services and emergency departments, between emergency departments and the general ward and ICU, and between general wards, ICUs, and the primary care team will help fill in some of the gaps that currently exist in the continuum of critical illness.

## Abbreviations

CPR: Cardiopulmonary resuscitation
CT: Computed tomography
ICU: Intensive care unit
MEWS: Modified Early Warning Score
NEWS: National Early Warning Score
qSOFA: Quick Sequential Organ Failure Assessment
RRS: Rapid response system
